# The Value of Electronic Medical Record Implementation in Mental Health Care: A Case Study

**DOI:** 10.2196/medinform.6512

**Published:** 2017-01-05

**Authors:** Sanaz Riahi, Ilan Fischler, Melanie I Stuckey, Philip E Klassen, John Chen

**Affiliations:** ^1^ Ontario Shores Centre for Mental Health Sciences Whitby, ON Canada; ^2^ Department of Psychiatry University of Toronto Toronto, ON Canada

**Keywords:** electronic health records, health information management, medical informatics, mental health, organizational innovation, psychiatry, quality improvement

## Abstract

**Background:**

Electronic medical records (EMR) have been implemented in many organizations to improve the quality of care. Evidence supporting the value added to a recovery-oriented mental health facility is lacking.

**Objective:**

The goal of this project was to implement and customize a fully integrated EMR system in a specialized, recovery-oriented mental health care facility. This evaluation examined the outcomes of quality improvement initiatives driven by the EMR to determine the value that the EMR brought to the organization.

**Methods:**

The setting was a tertiary-level mental health facility in Ontario, Canada. Clinical informatics and decision support worked closely with point-of-care staff to develop workflows and documentation tools in the EMR. The primary initiatives were implementation of modules for closed loop medication administration, collaborative plan of care, clinical practice guidelines for schizophrenia, restraint minimization, the infection prevention and control surveillance status board, drug of abuse screening, and business intelligence.

**Results:**

Medication and patient scan rates have been greater than 95% since April 2014, mitigating the adverse effects of medication errors. Specifically, between April 2014 and March 2015, only 1 moderately severe and 0 severe adverse drug events occurred. The number of restraint incidents decreased 19.7%, which resulted in cost savings of more than Can $1.4 million (US $1.0 million) over 2 years. Implementation of clinical practice guidelines for schizophrenia increased adherence to evidence-based practices, standardizing care across the facility. Improved infection prevention and control surveillance reduced the number of outbreak days from 47 in the year preceding implementation of the status board to 7 days in the year following. Decision support to encourage preferential use of the cost-effective drug of abuse screen when clinically indicated resulted in organizational cost savings.

**Conclusions:**

EMR implementation allowed Ontario Shores Centre for Mental Health Sciences to use data analytics to identify and select appropriate quality improvement initiatives, supporting patient-centered, recovery-oriented practices and providing value at the clinical, organizational, and societal levels.

## Introduction

Electronic medical records (EMRs) have been adopted in health care facilities around the world with the purpose of providing clinical, organizational, and societal value [1]. According to the Health Information Management Systems Society (HIMSS) EMR adoption model, a hospital can range from stage 0, which denotes zero to very minimal electronic record function, to stage 7, signifying a complete EMR with data analytic function that is meaningfully used to improve care [2]. In Canada, EMR adoption is low with 62.7% of Canadian health care facilities at stages 2 to 3 compared with 63.0% of American facilities at stages 5 to 6 in 2015 [2]. Although beyond the scope of this paper, there are many differences between the American and Canadian health care systems, including funding and incentive structures, that explain these differences. There are a number of benefits to EMR systems that are not being realized in Canada due to lack of adoption. For example, EMRs provide organizational and societal value by increasing research ability, averting costs, improving legal and regulatory compliance, increasing career satisfaction, and monitoring public health [1]. Clinically, EMRs have the potential to improve health care delivery across the 6 dimensions of quality care (safety, effectiveness, efficiency, patient-centered, timely, equitable [3]), although most literature has reported outcomes related to safety, effectiveness, and efficiency [1,4-9]. There are also a number of disadvantages of EMRs, which are likely significant factors explaining the low adoption rates in Canada. These include the large upfront investment to cover costs, potential interruptions in workflow with system initiation, privacy concerns, limitations to physicians’ autonomy, and unintended adverse outcomes [1,10]. Proper planning and support, however, can mitigate these challenges.

Ontario Shores Centre for Mental Health Sciences (Ontario Shores) implemented an EMR in 2009 and has since been committed to its meaningful use. In October 2014, Ontario Shores became the first Canadian hospital to achieve HIMSS Stage 7 designation and in October 2015, the first to achieve the HIMSS Nicholas E Davies Enterprise Award of Excellence (for the outstanding achievement of organizations who have used health information technology to substantially improve patient outcomes while achieving return on investment). Ontario Shores’ EMR adoption journey is unique in that the EMR needed not only to provide the basic medical and business intelligence functions but also to be customized to suit the specific needs of mental health care. Most of the benefits of EMRs reported are from general health care settings. Mental health care facilities have unique needs for monitoring quality care. Perhaps most notably, rather than following a medical model of care, many mental health care facilities have adopted a recovery model of care [11-13], which is strongly focused on patient-centered care and involves collaborative treatment planning between patients and clinicians to support the patients’ personal journeys toward achieving their individual goals. These goals may be related to symptom control or to promoting a meaningful, satisfying, hopeful, and contributing life, thereby minimizing limitations caused by illness. This case study reports on the implementation and customization of an EMR for a specialized, recovery-oriented mental health care facility. The objective of this evaluation was to determine the value that the EMR brought to the organization by examining the outcomes of quality improvement initiatives driven by the EMR.

## Methods

### Setting

Ontario Shores (Whitby, Ontario, Canada) is a public teaching hospital specializing in comprehensive mental health and addiction services for those with complex, serious, and persistent mental illness. The facility has 16 specialized inpatient units and extensive outpatient and community services serving a total regional population of approximately 2.8 million. The organization is staffed by approximately 1300 employees with 326 inpatients beds and approximately 60,000 annual outpatient visits. Ethical approval for this corporate evaluation study was not required by the institution as per Tri-Policy Statement guidelines.

### Implementation

In 2007, Ontario Shores began its journey to implement a fully integrated EMR system. The project consisted of two main steps: first, the organization-wide implementation of the EMR and second, the EMR modules customized to support recovery-oriented mental health care practices and data used to identify and evaluate quality improvement initiatives. A number of evidence-informed strategies were incorporated in the implementation plan [14]. Additionally, it was recognized that staff behavior change would be important to the successful adoption of new practices. Hence, change models were incorporated in the implementation plan. At the onset, Kotter’s change model [15] was followed to engage staff throughout the process. The Canada Health Infoway change management framework [16] was adopted when it was released.

### Step 1: Electronic Medical Record Implementation

#### Overview

Readiness work prior to building and launching the EMR system was emphasized. The clinical informatics portfolio was strategically aligned under the professional practice umbrella to ensure that clinical practice was the focus of all functionality, design, and development of the EMR, such that the technology would enable but not drive practice. A governance model was created, which necessitated engagement of key clinical stakeholders including a physician champion [17]. A key success in the readiness phase and overall implementation was early and continuous engagement of clinical staff, who were involved in vendor and system selection (Meditech 6.0, Westwood, MA, USA) and working groups along with information technology, clinical informatics, and professional practice specialists. Working groups designed new documentation forms that conformed to evidence-based guidelines and future state processes. New workflows were initiated with paper-based forms prior to EMR implementation to allow staff to become comfortable with new processes using a familiar charting medium.

#### Staff Training

Basic computer skill training was offered to all clinical staff. Intensive EMR-specific training sessions, led by professional practice, were required for all nursing staff, allied staff, and physicians. Content was based on an “a day in the life of” concept and walked the clinicians through EMR documentation from the beginning to the end of shift. Real-time practice with a test patient was incorporated to increase understanding and retention. Super users for training and on-unit support were important facilitators for successful implementation.

#### Go Live

Go live occurred in “Big Bang” fashion for all departments as applicable modules were ready (Table 1). Go live for financial, admissions, pharmacy, material management, human resources, and staffing and scheduling modules occurred from October to December 2009. Go live for all inpatient services occurred in October 2010 and for all outpatient services in August 2011. All advanced clinical applications including computerized physician order entry, electronic medication administration record and bedside medication verification, plan of care, patient care system, imaging and therapeutic services, laboratory, and physician care manager were included in the initial launch.

**Table 1:** Timeline for electronic medical record implementation, quality improvement initiatives, and significant milestones.

Phase	Period	Milestone
**Readiness phase**		
	2007/08	Request for information vendor shortlist and request for proposal. Business case approved. Contract signed. Project resource plan developed and core team assembled.
	2007/08	Device selection. Vendor demonstrations and evaluation.
	September 2008	Readiness work.
	March 2009	Design of new clinical forms.
**Implementation**		
	August 2009	Completion of Advanced Clinical System Readiness Assessment.
	October 2009	Go live: financial, admissions, pharmacy, material management modules.
	December 2009	Go live: human resources, staffing and scheduling.
	March 2010	Implementation of paper forms with new workflows.
	Summer 2010	Basic computer skills training offered.
	September 2010	Inpatient clinical staff EMR^a^-specific training.
	October 2010	Inpatient go live: Go live with EMR advanced clinical applications, patient care system, imaging and therapeutic services, laboratory, and physician care manager.
	December 2010	Data repository functional.
	June 2011	Outpatient clinical staff EMR-specific training.
	August 2011	Outpatient go live: Go live with all applicable modules for outpatient services.
**Customization and quality improvement**		
	2011/12	Full system upgrade plus integration of the Resident Assessment Instrument—Mental Health and optimization of restraint minimization practices and documentation.
	2012/13	Achievement of HIMSS^b^ stage 6 (June 2012). Plan of care optimization.
	2013/14	Full system upgrade plus implementation of integrated assessment record, business intelligence, and smoking cessation module. Optimization of outpatient and laboratory modules.
	2014/15	Achievement of HIMSS stage 7 (October 2014). Implementation of CPGs^c^ for schizophrenia, level of care utilization system, front-end speech recognition, Ontario Common Assessment of Need, infection prevention and control surveillance status board; optimization of quality risk management. Launch of patient portal for patient engagement and self-management.
	2015/16 (to date)	Achievement of HIMSS Nicholas E Davies Enterprise Award of Excellence (October 2015). Technology-sharing partnership with other mental health facilities. EMR optimization through evaluation and enhancement of existing modules. Participation in health information exchange initiatives.

^a^EMR: electronic medical record.

^b^HIMSS: Health Information Management Systems Society.

^c^CPG: clinical practice guideline.

### Step 2: Customization, Sustainability, Optimization, and Quality Improvement

In addition to the standard modules, a number of modules were created to support recovery-oriented care. Once modules were implemented, data mining was used to identify the need for quality improvement initiatives as described below.

### Standard Modules

#### Closed Loop Medication Administration

The computerized physician order entry, electronic medical administration record, and bedside medication verification modules were standard and included in the initial launch. An adherence rate of 95% was needed in order to qualify for HIMSS stage 7 designation. An audit of bedside medication verification data showed that medication and patient scan rates were below this target. Letters of expectation communicating the standards of practice and importance of meeting target adherence levels in regard to safety were issued to all nursing staff whose adherence was lower than 90% over a 6-month period. A working group with point-of-care nurses was deployed to identify and remedy practice and process issues that were contributing to the low adherence rates.

#### Business Intelligence

Data mining has resulted in the evolution of a business analytics culture. With the implementation of a business intelligence tool, opportunities were created to build data models and engage in data analytics. Summary data for key performance indicators were provided on a dashboard with drill-through functionality. Detailed information related to program needs was accessible to leadership staff to help guide decision-making processes related to operations.

### Mental Health Specific Modules

#### Resident Assessment Instrument—Mental Health

The Resident Assessment Instrument—Mental Health (RAI-MH), which is necessary for mandatory reporting to the ministry in the province of Ontario, Canada, was historically completed in another solution. With the implementation of the EMR, this mandatory assessment has been integrated in the EMR, and clinician daily documentation assists in completing this assessment. The RAI-MH is an interdisciplinary tool in the EMR with alerts to notify the clinical team of any missing measures and timely completion.

#### Plan of Care

The plan of care is a patient-centered treatment and discharge plan that informs care with the intention of ensuring a seamless transition and continuity of care between service providers. The patient, substitute decision maker, and family caregivers (if applicable) work collaboratively with the interprofessional team to develop the plan of care. The patient’s values, strengths, goals, and vision are provided by the patient and family while the assessment, medical history, and behavioral profiles are added by the interprofessional team. The plan of care contains a number of themes that need to be addressed in mental health care (such as harm to self, illness management, exercise and nutrition, and leisure and education). On admission, relevant themes are selected and goals are developed in collaboration between the patient, relevant family, and clinicians.

Important to recovery-oriented treatment, each plan of care includes a recovery plan with 3 sections: patient story (subjective information gathered from the patient upon admission and on an ongoing basis to explore personal values, interests, cultural and religious practices, support systems, and personal views on hospitalization and treatment), crisis prevention plan (information regarding a patient’s unique behavioral pattern, antecedents to maladaptive behaviors, de-escalation preferences, strategies, and intervention techniques), and sensory diet (sensory modalities that provide patients with information for recognizing and reducing their level of self-perceived distress and for modulating and learning to self-regulate their mood and behaviors).

#### Restraint and Seclusion

To align practices with recovery values, a policy supporting least restraint and seclusion was implemented [18]. Decision support was embedded directly within the physician orders for restraint and seclusion, and appropriate documentation of best practices were reflexed for clinical staff. Specifically, clinicians were alerted to complete a reassessment hourly for the duration of the event. Prompts were included to encourage clinicians to engage the patient with alternative interventions with the goal to reduce their duration of time in restraint or seclusion. Additionally, prompts were built in to remind clinicians to update patient de-escalation preferences in the plan of care following the event with the intention of preventing future incidents. A section was added to the module to document the patient and clinician debrief following the incident. Daily, monthly, and quarterly restraint data were reviewed by the senior management team, clinical leadership, and staff. Data were used to guide and facilitate meaningful, nonpunitive discussions with unit staff and to inform future practices aimed at minimizing restraint and seclusion use.

#### Clinical Practice Guidelines for Schizophrenia

Reflex orders and decision support were embedded in electronic documentation templates to prompt clinicians to follow evidence-based practices for the assessment and treatment of schizophrenia [19]. Since many standard practices at Ontario Shores were already aligned with the clinical practice guidelines (CPGs) for schizophrenia, the primary additions to the EMR were for antipsychotic prescribing (to promote adherence to antipsychotic monotherapy), metabolic monitoring, and referral to cognitive behavioral therapy for psychosis (CBT-P) and vocational rehabilitation. A tracking template was created for psychopharmacologic trials to document historical pharmacological trials, side effects, and outcomes with the intention of providing physicians with information that may help make decisions about antipsychotic prescribing. Exception handling was also added to require physicians to select a reason for ordering polypharmacy to prompt reflection and consideration for monotherapy. Since metabolic abnormalities are common side effects of antipsychotic medications, a reflex order set was created for metabolic monitoring triggered by the input of an order for antipsychotic medication. The order set included annual glucose and lipid panels and monthly blood pressure, body weight, and waist circumference measurement as per guidelines. Reflex orders were also created to trigger referral to CBT-P and vocational rehabilitation services upon entering a diagnosis of schizophrenia.

To support adherence to CPG practices, adherence data were extracted from the EMR and shared with physicians, clinical practice leaders, leadership staff, and clinical teams using clinical scorecards built into a business intelligence tool. When adherence to guidance was suboptimal, quality improvement projects were initiated.

#### Infection Prevention and Control Surveillance Status Board

Due to an increasing number of outbreak days (ie, total number of days in which an outbreak was declared on any unit in the organization) at Ontario Shores, EMR functionality was leveraged to improve symptom surveillance and optimize the processes for initiating precautions and monitoring symptoms. The symptom surveillance assessment and communication tool was digitized and a field was added to allow nursing staff to initiate precautions electronically without consulting physicians. The infection prevention and control surveillance status board allowed infection prevention and control practitioners to monitor symptoms from any EMR-enabled workstation in the hospital, quickly identifying any need for follow-up.

#### Drug of Abuse Screening

In response to increased laboratory costs, a review of the quantity and expense of tests ordered was performed via the EMR. An expensive, broad-spectrum drug screen was being preferentially ordered rather than the drug of abuse screen. Discussion between the physician advisory group and the medical advisory committee established that the less expensive drug of abuse screen met clinical needs in the majority of cases. Thus, decision support was built into the EMR to support the physician order of the drug of abuse screen and protocols were added to identify the components of each test.

#### Patient Portal

In alignment with the recovery model, Ontario Shores’ health check portal was implemented in 2014 to enhance patient access to their personal health information and promote patient activation and partnership between patients and clinicians. The portal was a built-in function of the EMR that was customized to Ontario Shores’ needs. Full details have previously been reported [20]. Briefly, upon portal enrollment, patients were able to access parts of their medical record, view upcoming appointments, and communicate with their clinician from any device with Internet connectivity. Patients could choose to share information with family or community support workers.

### Data Analysis

Monthly adherence to patient and medication scan rates was retrieved from the EMR from July 2013 to March 2015 (9 months of monitoring adherence prior to and 12 months of follow-up after quality improvement intervention). Monthly adherence to CPG practices (antipsychotic monotherapy, metabolic monitoring, and referral to CBT-P and vocational services) was retrieved from the EMR for the month prior to and 12 months following CPG implementation (March 2014 to March 2015). Restraint and seclusion rates were used as a proxy for adherence to least restraint practices under the assumption that adherence to recovery-oriented practices would reduce the number of restraint and seclusion incidents. The number of restraint and seclusion incidents was retrieved from the EMR from April 2011 to March 2014. Finally, outbreak days were used as a proxy for adherence to infection prevention and control practices with the assumption that if procedures were followed, transmission rates, and, therefore, number of outbreak days would be reduced. Data were compiled from infection prevention and control documentation (April 2013 to March 2015), as these data were not entered into the EMR prior to implementation. For all other initiatives, anonymous summary data were retrieved from the corporate database. Descriptive analysis was used to examine changes in practice (Excel spreadsheet, Microsoft Corp). A crude return on investment summary was completed by documenting the initial investment, operational costs, and both hard and soft return on investments.

## Results

### Standard Modules: Closed Loop Medication Administration

**Figure 1:**
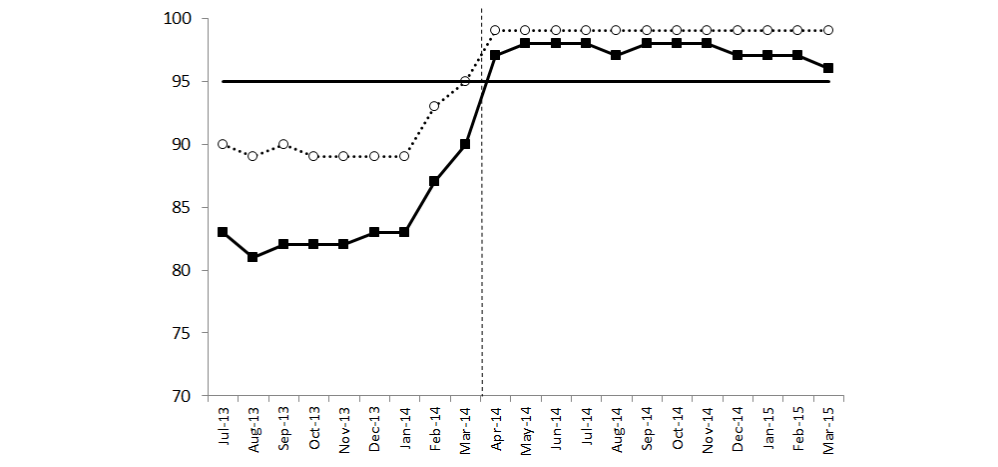
Patient and medication scan rates. Closed squares, solid line: medication scan rate; open circles, dotted line: patient scan rate; solid horizontal line: target 95% adherence; dashed vertical line: implementation of quality improvement initiative.

Prior to the quality improvement initiative, patient and medication scan rates ranged from 80% to 95%. Following distribution of letters of expectation and follow-up with clinical managers (March 2014), scan rates were maintained above 95% (Figure 1). This was accompanied by a reduction in the number of moderate adverse drug events from 5 in 2011 (initial implementation) to 2 in each of 2012 and 2013 (prior to achievement of acceptable adherence rates) and 1 in 2014 (acceptable adherence rate).

### Specialty Mental Health and Recovery Modules

#### Clinical Practice Guidelines

In the 12 months following CPG implementation, modest improvements were realized in CPG adherence [21]. Adherence to CBT-P and vocational rehabilitation guidance was increased from 6.5% to 11.4% and 36.6% to 49.1%, respectively. Adherence to antipsychotic monotherapy guidance increased initially from 53.4% to 62.7% but fell back to 55.1% by 12 months. Adherence to metabolic monitoring increased slightly from completing 76.7% of all required metabolic measurements to 81.6% [21].

#### Restraint and Seclusion

There was a 19.7% decrease in the number of restraint and seclusion incidents, a 42.3% decrease in the total restraint and seclusion hours, and a 38.9% decrease in the average hours per restraint or seclusion from the 2011/2012 fiscal year to the 2013/2014 fiscal year [18].

### Custom Modules Developed by Ontario Shores: Infection Prevention and Control

**Figure 2:**
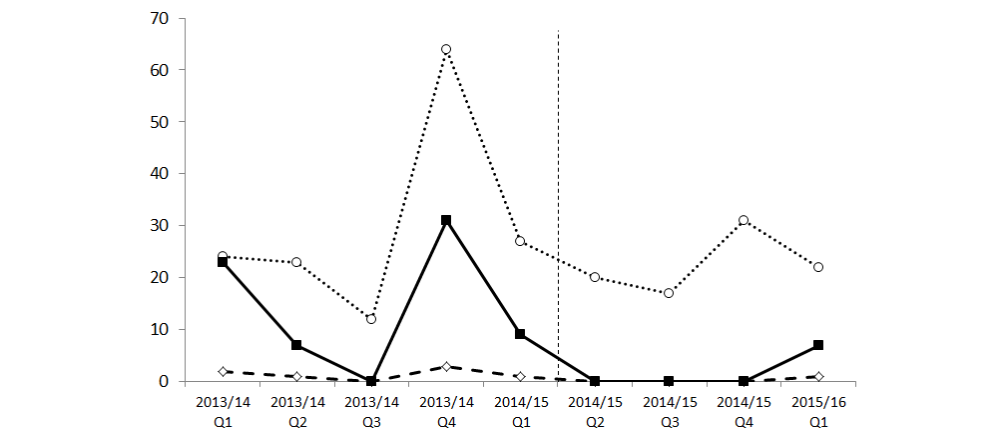
Number of precautions, outbreaks and outbreak days. Closed squares, solid line: outbreak days; open circles, dotted line: precautions; open diamonds, dashed line: outbreaks; dashed vertical line: implementation of infection prevention and control surveillance status board.

There were 7 outbreak days in the year following infection prevention and control surveillance status board implementation compared to 47 outbreak days in the previous year (Figure 2).

### Return on Investment

As shown in Table 2, after EMR implementation in 2009/2010, costs were recovered by year 2013/2014. Much of the crude cost-savings were attributed to reduction in materials and salaries needed to support paper charting. Significant savings were also realized from the reduction in restraint and seclusion events following implementation of the restraint and seclusion modules in the EMR.

**Table 2:** Crude return on investment summary for electronic medical record implementation. All currency is presented in Canadian dollars.

			Initial	2010/11	2011/12	2012/13	2013/14	2014/15
**Capital/one-time investment/implementation**								
	Initial investment and implementation cost		6,546,274	—	—	—	—	—
	Staffing		4,135,900	—	—	—	—	—
	Total initial investment		10,682,174	—	—	—	—	—
**Operational costs (incremental)**								
	Incremental IT/CI^a^ staffing		—	563,768	871,960	881,933	873,036	864,333
	Maintenance and support—EMR^b^		—	173,093	289,059	270,811	329,293	333,388
	Incremental hardware, software, and licensing		—	24,864	14,215	77,512	17,264	428,481
	Total expenses		—	761,725	1,175,234	1,230,256	1,219,594	1,626,202
**Hard return on investment**								
	**Savings/efficiencies**							
		HIM^c^ paper chart savings	—	15,000	20,000	25,000	25,000	25,000
		HIM chart control staff Reduction	—	—	185,865	200,630	209,831	341,885
		Transcription service savings	—	—	101,280	107,955	97,969	118,071
		Lab test utilization	—	—	—	—	16,372	16,372
		Reduced clerical and admin overhead	—	150,000	153,000	156,060	159,181	162,365
		Reduction in medication cost due to unit dose and utilization	—	196,078	200,000	204,000	208,080	212,242
		Staff savings from restraint and seclusion prevention	—	—	—	—	776,633	666,957
		Reduction in antipsychotics due to CPG^d^	—	—	—	—	—	12,240
	**Revenue/incentives**							
		Grants	—	—	—	—	50,000	216,563
**Soft return on investment**								
	**Cost savings/avoidance**							
		Adverse drug event	—	—	—	38,100	38,100	50,800
		Annual benefits	—	361,078	660,145	731,745	1,581,166	1,822,494
		Total annual cash flow	(10,682,174)^e^	(400,647)^e^	(515,0789)^e^	(498,511)^e^	361,573	196,293

^a^IT/CI: information technology/clinical informatics.

^b^EMR: electronic medical record.

^c^HIM: health information management.

^d^CPG: clinical practice guideline.

^e^Parentheses indicate deficit.

## Discussion

### Overview

Implementation of a fully integrated EMR and the use of data analytics to identify quality improvement projects provided value at the clinical, organizational, and societal levels. Customization for mental health care facilitated the adoption of recovery-oriented practices. One of the strengths of this project was that corporate data were used to capture the outcomes of the relevant population in its entirety rather than consenting a subset of participants, which may have introduced selection bias because more motivated and well patients would be more likely to volunteer to participate. Additionally, this project incorporated a number of implementation facilitators identified in the literature, such as active involvement and support of management, inclusion of clinical staff in the implementation and decision-making processes, end-user training and real-time support, and the identification of physician champion and point-of-care staff champions to provide support to peers [14]. Importantly, it was recognized that implementation was not purely a technical project and that behavior change of staff would be a critical element to the success of the project [14]. Relevant change models or frameworks were embedded in the implementation plan to guide the behavior change process [15,16].

### Comparison With Prior Work

The integration of clinical decision support tools into standard workflows was an important facilitator to the adoption of new workflows [6-9] and has been identified as particularly important to the adoption of evidence-based medicine [8,22]. Our CPG for schizophrenia adoption strategy relied heavily on clinical decision support. Adherence to practices, however, was not greatly changed, with the exception of referrals to CBT-P and vocational rehabilitation services [21]. Adherence to metabolic monitoring, for example, only increased slightly from 76.7% in the month prior to CPG implementation to 81.6% at 1-year follow-up [21]. This is in contrast to a previous study, which showed that when clinical decision support to alert the need for metabolic monitoring was used, the odds were 3.51 times greater that clinicians would complete the recommended measurements compared to a control group [7]. Differences may be due to baseline adherence, which was only around 3% [7] compared to our baseline of 76.7% [21]. As Ontario Shores adopts and integrates other CPGs within the EMR (eg, dementia, depression), appropriate coordination and use of clinical decision support will be required to maintain patient safety and a patient-centered approach to care. Barriers contributing to the failure of clinical decision support to aid in adoption include perception of usefulness and over-triggering [8]. Thus, careful planning and consideration is needed to optimally integrate clinical decision support in the EMR as functions become more complex. Indeed, it has been identified as an important component to appropriately integrate patient-centered approaches, combinations of CPGs, self-management interventions, and continuity of care in patients with multiple morbidities [23]. These may be especially important in recovery-focused mental health care to support patients who may have multiple diagnoses—both psychological and physical—and be working toward achieving individualized goals on their recovery journey.

Careful implementation of the EMR and additional modules resulted in clinical, organizational, and, potentially, societal value. Clinical value was seen across the 6 dimensions of quality care [3]. Notably, patient safety was improved through the reduction in restraint, seclusion, and associated adverse events; reduced infection transmission; and the prevention of medication errors from reaching the patient. Other studies examining the impact of EMRs in acute and general health care settings have shown that well-planned and implemented systems have reduced mortality and medical complications [4] but simple implementation has shown mixed results [1]. As in this study, others have demonstrated reduced medical errors and redundant diagnostic tests [5,24,25], thereby increasing treatment effectiveness and care efficiency along with safety. Implementation of CPGs ensured that all patients diagnosed with schizophrenia were provided the full spectrum of evidence-based assessments and treatments [6,7,9], ensuring the timely and equitable delivery of services while also contributing to improvements in the 3 aformentioned dimensions of quality care.

A number of initiatives implemented through the EMR to improve patient-centered care are poorly described in the literature but especially important to recovery-oriented mental health care [11-13]. The plan of care was implemented to lead clinicians through a method of collaborative treatment planning with patients and their families. In accordance with recovery principles, patient goals, strengths, and treatment preferences were documented with the intention of using those at the basis for planning care. The plan of care is reviewed once per shift to encourage staff to consider patients individually throughout their treatment. Modules for restraint and seclusion included patient de-escalation preferences and reflex orders to ensure timely debriefing following events with the intention of repairing the therapeutic relationship, which is often compromised [26]. Restraint data were reviewed extensively and informed initiatives implemented as Ontario Shores strove to minimize restraint and seclusion as per best practice guidelines. Ontario Shores’ health check portal was another important patient-centered initiative enabled by the EMR. It promoted patient activation by enabling patients to access their personal health information. A full benefits evaluation of the portal was previously reported [20] that showed improvements in patient activation and recovery over the year following implementation. Notably, a subset of patients who responded to a survey reported an increased sense of autonomy [20], which is an important component to mental health recovery [11-13]. Indeed, the EMR has played an important role in the incorporation of recovery-oriented services into practice and will remain a central component of future initiatives to promote patient-centered recovery.

Organizational and societal value was seen through the standardization of workflows, facilitating the fulfillment of legal and regulatory obligations. A benefits evaluation of the patient portal showed improved administrative efficiencies and productivity in the year following compared to the year prior to portal implementation [20]. Internally, the EMR data were presented to highlight trends, providing information for clinicians to support informed clinical decision making at the direct care level. Additionally, discrete data created opportunities to evaluate adherence to and effectiveness of treatment and to select appropriate quality improvement initiatives. An overall culture change has been realized along with greater efficiencies for clinical and nonclinical roles. Furthermore, the potential of the EMR to support research has been recognized, and it will play a strong role in the future.

It should be noted that a significant upfront monetary investment was needed. Similar to other studies, cost benefits were not realized immediately [27] but were seen after the EMR data were used to identify the need for and support the implementation of quality improvement initiatives. Most of the direct cost savings (Table 2) are a result of reduced costs in the health information management department, including transcription cost savings exceeding Can $425,000 since 2011/2012 and the reduction of supplies associated with paper filing. Paper communications with external entities and patients have been reduced through use of the health information exchange and health check portal, respectively, decreasing the preparation time for release of information. Many manual processes related to the collection of data for internal and external reporting, such as wait time data and workload statistics, have been eliminated, and the turnaround time has significantly decreased. Cost reductions were realized with decreased restraint and seclusion events and reduced antipsychotic medication prescribing (Table 2). It should be noted that a simple return on investment calculation for this mental health facility may underestimate the full benefit of EMR implementation. For example, while implementation of metabolic monitoring according to CPGs provides no cost savings to Ontario Shores, the anticipated prevention or delay of onset of cardiovascular diseases and type 2 diabetes mellitus has the potential to reduce the economic burden on the Ontario health care system. The EMR also enables health information exchange initiatives to integrate electronic patient information from across the care continuum to improve timely access at the point of care, ultimately improving the patient and clinician experience and reducing overall costs to the health care system.

The EMR has enabled a number of new initiatives at Ontario Shores. Recently (November 2016), a partnership was formed with another mental health facility to create a shared EMR using a single system and database. The purpose of this partnership is to continue to improve clinical outcomes, advance evidence-based practices and clinical standards, advance mental health research, and realize cost effectiveness and efficiencies. Future evaluation and study will be needed to determine the effectiveness of this partnership, which may set the stage for increased technology-sharing partnerships to increase EMR adoption in Canada.

### Limitations

Despite the above-mentioned strengths, this project was not without limitations. Since EMR implementation and modules were designed to optimize recovery-based practices at a specialized, tertiary care mental health facility, results may not be generalizable to all other settings. However, the framework and examples should provide a guideline for leveraging EMR functionality to optimize practices. Since this case study reports organizational practices, outcomes were limited to metrics available through corporate data. As such, many outcomes are proxy measures of adherence to practices. Explanatory variables such as staff perspectives were not collected and could not be included in this study. As Ontario Shores is continually striving to improve the quality of care, a number of concurrent non–EMR-based projects were initiated during the same time period. Therefore, some of the improvements may not have been directly related to the EMR or may have been further improved by other initiatives. Clear benefits of the EMR have, however, been described.

### Conclusions

This case report described the implementation of an EMR system in a tertiary-level mental health care facility to increase clinical, organizational, and societal value. Customization for mental health services enabled the provision of recovery-oriented services to patients overall improving delivery across the 6 dimensions of quality care. Future initiatives to further leverage the EMR potential for evaluation, quality improvement, research, and collaboration are underway.

## Abbreviations

CBT-P: cognitive behavior therapy for psychosis
CI: clinical informatics
CPG: clinical practice guideline
EMR: electronic medical record
HIM: health information management
HIMSS: Health Information Management Systems Society
IT: information technology
Ontario Shores: Ontario Shores Centre for Mental Health Sciences
RAI-MH: Resident Assessment Instrument—Mental Health
